# THE IMPACT OF AGING ON ATTITUDES TOWARD WEIGHT CHANGE AND BODY IMAGE AMONG ADULTS LIVING WITH HIV

**DOI:** 10.1093/geroni/igad104.1350

**Published:** 2023-12-21

**Authors:** Jennifer Kaufman, Yiyi Wu, Jasmine Manalel, Ethan Fusaris, Arlene Correa, Jerome Ernst, Mark Brennan-Ing

**Affiliations:** Brookdale Center for Healthy Aging, Hunter College, City University of New York, New York City, New York, United States; Hunter College, City University of New York, New York City, New York, United States; Hunter College, New York City, New York, United States; Amida Care, New York, New York, United States; Brown University, Providence, Rhode Island, United States; Amida Care, New York, New York, United States; Hunter College, CUNY, New York City, New York, United States

## Abstract

Some newer HIV antiretroviral therapy regimens have been associated with weight gain in populations at risk for obesity. This study examined perspectives on weight change and body image among people living with HIV and the extent to which age and gender have an impact on those attitudes. We conducted interviews with 61 individuals in a Medicaid managed care plan. The age range was 23 to 65 years; 49% were aged 50 or older. The sample was 56% non-Hispanic Black and 31% Hispanic. About half were cisgender men, 36% were cisgender women, and 13% were transgender/nonbinary individuals. Many participants had weight gain they associated with HIV medications. While experiences varied widely, we found that older and younger participants offered some different perspectives. Younger people, including transgender individuals, more often welcomed weight gain, associating it with better health. Participants aged 50 and older sometimes saw weight gain as a natural part of aging, and spoke of acceptance, whereas younger participants tended to focus on their appearance. Among those with unwanted weight gain, older participants expressed concerns about its negative effect on mobility and comorbid health conditions. Some older women in particular struggled with improving their diets. Healthcare providers should consider these different perspectives and the various challenges individuals face associated with age when advising patients with HIV about weight management. For providers working with older patients with HIV, specific considerations of how increased weight affects mobility and risk for comorbid conditions are warranted.

